# Highly Bactericidal Macroporous Antimicrobial Polymeric Gel for Point-of-Use Water Disinfection

**DOI:** 10.1038/s41598-018-26202-0

**Published:** 2018-05-21

**Authors:** Amit Kumar, Cyrille Boyer, Leena Nebhani, Edgar H. H. Wong

**Affiliations:** 1 Department of Materials Science and Engineering, Indian Institute of Technology Delhi, Hauz Khas, New Delhi, 110016 India; 2 Centre for Advanced Macromolecular Design (CAMD) and Australian Centre for NanoMedicine (ACN), School of Chemical Engineering, UNSW Australia, Sydney, NSW 2052 Australia

## Abstract

Access to clean and safe water supply remains inadequate in many developing countries. One of the key challenges is to remove pathogenic bacteria from the water supply via effective water disinfection technologies to prevent the spread of diseases and to ensure the safety of consumers. Herein, a highly effective point-of-use (on-demand) water disinfection technology, in the form of a polymeric scaffold called macroporous antimicrobial polymeric gel (MAPG), is demonstrated. MAPG is easy to fabricate, completely organic and possess inherent antimicrobial property which makes it non-reliant on inorganic compounds such as silver where the long-term toxicity remains unknown. MAPG is highly bactericidal and can disinfect bacteria-contaminated water (ca. 10^8^ CFU mL^−1^) at a capacity of about >50 times the mass of the organic material used, inactivating >99% of both Gram-negative and Gram-positive bacteria including *Escherichia coli*, *Vibrio cholerae* and *Staphylococcus aureus* within 20 minutes of treatment. When fabricated in a syringe, MAPG eliminates *E. coli* from contaminated water source by >8.0 log_10_ reduction in bacteria counts (i.e., no viable bacteria were detected after treatment), and the syringe can be reused multiple times without losing potency. The MAPG technology is not only restricted to water disinfection but may also be applicable in other bacteria inactivation applications.

## Introduction

Despite the advances in water purification technologies, access to clean and safe fresh water supply remains inadequate in some major populations of developing countries^1,2^. A pressing challenge in water purification is bacterial disinfection^3^, as ineffective treatment results in the presence of pathogenic bacteria in the water supply which then leads to the spread of diseases. To make matters worse, the rise of multidrug-resistant bacteria in recent years increases the difficulty in combating these microorganisms^4,5^. Common purification devices mainly utilize membrane filtration to simply isolate the bacteria by size differences which do not necessarily result in bacteria cell death^6,7^. These devices are prone to fouling on the surface because of biofilm formation and are costly and difficult to clean to be considered reusable^8,9^. Furthermore, the operation of membrane filtration devices requires high energy consumption^10^. Thus, new strategies of water disinfection that are economical and efficient in inactivating bacteria in the water supply will be worthwhile exploring.

The incorporation of inorganic nanoparticles (e.g., silver) or chlorine-releasing precursors in polymeric substrates/materials that are typically found in water disinfection devices are some of the most widely reported methods for inactivating bacteria in the water supply^11–14^. These materials generally exhibit moderate to good antibacterial activity. However, there are several drawbacks associated with them. For instance, the release of silver ions into the water supply poses potential toxicity to the consumers while the fabrication of these materials can be costly and tedious. Additionally, these devices will experience the inevitable loss of activity once the active compounds have been released. Other potential strategies may include the development of polymeric materials, such as hydrogels/scaffolds, that inherently possess antimicrobial properties (i.e., the material itself inactivates adhering bacteria upon contact)^15–19^. Although the antimicrobial polymer scaffolds reported so far are more suited for biomedical applications (e.g., wound-healing) than water disinfection purposes because of their limited ability to disinfect only small volumes of water, this class of material represents a promising approach for safer point-of-use water disinfection applications given that the antimicrobial activity is not imparted through leaching of potentially toxic reagents into the water supply.

In this study, we report the development of a novel polymeric material which possess excellent inherent antimicrobial activity, termed macroporous antimicrobial polymeric gel (MAPG), that can effectively disinfect bacteria-contaminated water >50 times the mass of the organic content material used. MAPG possess broad-spectrum antimicrobial activity, capable of inactivating >99% of both Gram-negative and Gram-positive bacteria including *Escherichia coli*, *Vibrio cholerae* and (methicillin-resistant) *Staphylococcus aureus* within 20 minutes of treatment. In addition, MAPG can be synthesized in a facile manner from affordable commercially available chemicals. Furthermore, we also demonstrate that MAPG can be fabricated as antibacterial syringes that can be recycled multiple times without losing potency. To the best of our knowledge, MAPG is one of the most potent antibacterial polymer gels ever developed that can potentially be translated to real world water disinfection applications. The excellent activity of MAPG owes to the combination of key two factors: i) the use of facially amphiphilic cationic polymers, and ii) the macroporous structure of the material. While the polymers are responsible for effecting bactericidal property by disrupting the bacteria membrane cell wall in the same vein as antimicrobial peptides and synthetic polymer mimics, the macroporous structure enables the gel to act like a sponge and adsorb the bacteria from the solution, thereby allowing for maximal contact between bacteria and polymer because of the high surface area within the gel internal structure. The platform technology developed herein is not only restricted to water disinfection but may also be applicable in other bacteria inactivation applications.

## Results

### Material Fabrication and Characterization

**Figure 1:**
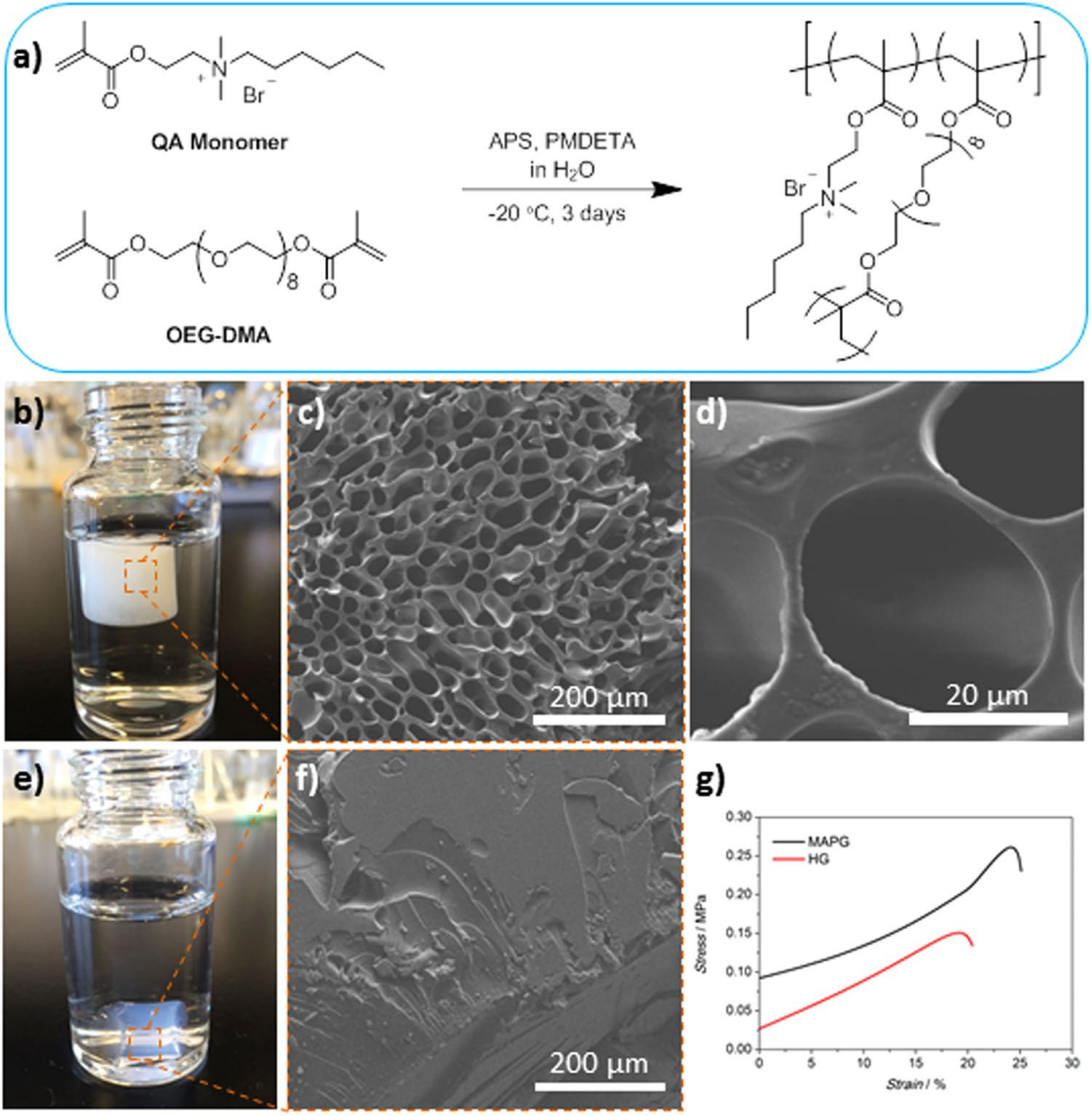
Synthesis of polymeric gels and their physical characteristics. (**a**) Schematic showing the synthesis of macroporous antimicrobial polymeric gel (MAPG) via free radical polymerization at subzero temperature. (**b**) Image of MAPG in water. (**c** and **d**) SEM micrographs of a swollen MAPG. (**e**) Image of hydrogel (HG) in water and (**f**) the corresponding SEM micrograph. (**g**) Stress vs strain curve of the gels during mechanical compression test.

To make MAPG, a quaternary ammonium (QA) methacrylate monomer that bears a hydrophobic n-hexyl tail was first synthesized (Fig. 1a). The monomer was chosen on the basis of our experience in developing synthetic antimicrobial polymers, where the judicious combination of cationic and hydrophobic groups is necessary to exert good antimicrobial activity^20,21^. Specifically, the n-hexyl group was selected as sufficient hydrophobicity is required to cause membrane disruption while the ammonium center enables for binding with the anionic character of the bacteria cell wall. The synthesis of the QA monomer via quaternization reaction between 2-(dimethylamino) ethyl methacrylate and 1-bromohexane proceeded in a straightforward fashion and can be performed easily at multigram scale. ^1^H and ^13^C NMR analysis confirmed successful monomer synthesis (Fig. S1, Supporting Information (SI)). To fabricate MAPG, the QA monomer was polymerized via free radical polymerization in the presence of a redox radical initiator (i.e., ammonium persulfate), coinitiator *N,N,N′,N″,N″-*pentamethyldiethylenetriamine (PMDETA) and a cross-linkable monomer (i.e., oligoethylene glycol dimethacrylate (OEG-DMA)) in water at subzero temperature (Fig. 1a). The macroporous structure of the gel is generated based on the formation of ice crystals which act as sacrificial templates within the aqueous polymerizable media at subzero temperature. This method of forming macroporous gels is called cryogelation^22–25^. The overall synthesis process (i.e., from monomer to gel generation) negates the involvement of elaborate lab setup or technique and requires only simple mixing of chemicals, hence making the whole process potentially viable for industrial scale preparation.

The formed MAPG is white in appearance and SEM analysis of the swollen wet gel confirmed the presence of a macroporous structure where the pore size is ca. 15–30 µm in diameter (Fig. 1b–d). Interestingly, MAPG has buoyancy in water which is due to the porous structure of the material. Compared to an analogous hydrogel (HG), which was prepared at room temperature and hence has no ice crystal templates to yield macroporous structure like MAPG, HG has no buoyancy in water (Fig. 1e). SEM analysis of the wet HG confirmed the non-porous structure of the gel (Fig. 1f). Furthermore, HG appears as a transluscent gel, unlike MAPG which is white and non-transparent. The difference in appearance between MAPG and HG is typical of common cryogels and hydrogels even though they consist of the same chemical composition^22,23^. In terms of swelling ability, MAPG has a higher water uptake than HG where the swelling degree (i.e., the weight ratio of a fully swollen gel to dried gel) for MAPG and HG is 9.5 and 4.5, respectively (Table 1). We also observed that the swelling rate of MAPG is quicker than HG. MAPG achieved a fully swollen state in ca. 10 seconds whereas HG required 500 seconds to be fully swollen (Fig. S2, SI). The superior swelling performance of MAPG is attributed to the porous structure of the material which enables the gel to absorb/uptake large amount of liquid.

**Table 1:** Physical properties and antimicrobial activity of polymer gels against various Gram-negative and Gram-positive bacteria.

Gel	*θ* ^a)^	*E*^b)^/kPa	Time^c)^/min	Log_10_ bacteria reduction in bulk water^d)^				
				*P. aeruginosa*	*E. coli*	*V. cholerae*	*S. aureus*	*MRSA*
MAPG	9.5	567 ± 74	20	4.7 (99.998)	5.0 (99.9991)	2.1 (99.26)	2.1 (99.28)	3.0 (99.89)
			60	6.5 (99.99997)	6.5 (99.99997)	4.1 (99.993)	>8.2 (100)	3.2 (99.93)
HG	4.5	683 ± 42	60	0.2 (37.5)	nd^e)^	nd	1.5 (96.8)	nd

^a)^Swelling degree (*θ*) determined as the weight ratio of swollen gel to dried gel; ^b)^Measured in swollen state; ^c)^Gel incubation time in bacteria-contaminated water; ^d)^The bacterial strains are *Pseudomonas aeruginosa* PAO1, *Escherichia coli* K12, *Vibrio cholerae* SIO, *Staphylococcus aureus* SA31, and methicillin-resistant *S. aureus* SA60. The number in parentheses indicates the percent bacteria reduction in bulk water compared to untreated controls; ^e)^nd indicates not determined.

The mechanical property of the swollen gels was subsequently determined via uniaxial compression tests. The stress-strain curve as shown in Fig. 1g indicated that both MAPG and HG have similar profile although HG suffered irreversible mechanical fracture when compressed to 19.5% strain of its initial length (at a maximum compression force of 160 ± 8 kPa) whereas MAPG can withstand compression of up to 24.5% strain (compression force of 280 ± 9 kPa). The Young’s modulus (*E*) of MAPG and HG are 567 ± 74 kPa and 683 ± 42 kPa, respectively (Table 1). In general, MAPG and HG which were made from QA and OEG-DMA monomers are mechanically tougher than other reported cryogels and hydrogels where the *E* values of these materials are typically in the range of 5–30 kPa^15,19^. Besides the covalently cross-linked networks afforded by the polymerization process which keep the polymers interconnected in a gel, we postulate that secondary hydrophobic interactions are present which endow the gels with higher mechanical toughness. Specifically, we believe that the hydrophobic n-hexyl groups from the QA monomer form non-covalent hydrophobic inclusions which further strengthen the gel network, akin to a double network hydrogel system^26,27^. This postulation was supported by the observation that gels made from QA monomer bearing a methyl group, which is far less hydrophobic than the n-hexyl group, are very unstable (easily breakable) and brittle compared to those made from n-hexyl-functionalized QA monomer.

### Broad-Spectrum Bacterial Disinfection of Contaminated Bulk Water

The antimicrobial activity of MAPG was assessed against different Gram-negative and Gram-positive bacteria, namely *Pseudomonas aeruginosa*, *Escherichia coli*, *Vibrio cholerae*, and *Staphylococcus aureus*. For this, the fully swollen gels were sliced into thin discs (ca. 15 × 2 mm and 200 mg in weight) and each placed in a well of a 24-well plate. Bacteria-contaminated water (1 mL of ca. 10^8^ colony forming units (CFU) mL^−1^) was added to the well and incubated for a predetermined time period (i.e., 20 and 60 min). The amount of viable bacteria that remained in the bulk water after treatment, denoted as ‘treated’ water, was determined via standard CFU analysis and compared to the untreated sample (i.e., the negative control). It is noteworthy that these bacteria (e.g., *E. coli* and *V. cholerae*) were selected on the basis that they are some of the most common bacteria found in contaminated water supply^28^. In addition, we have also included methicillin-resistant *S. aureus* (MRSA)^29^ in our study to ascertain the ability of MAPG to combat antibiotic-resistant strains.

**Figure 2:**
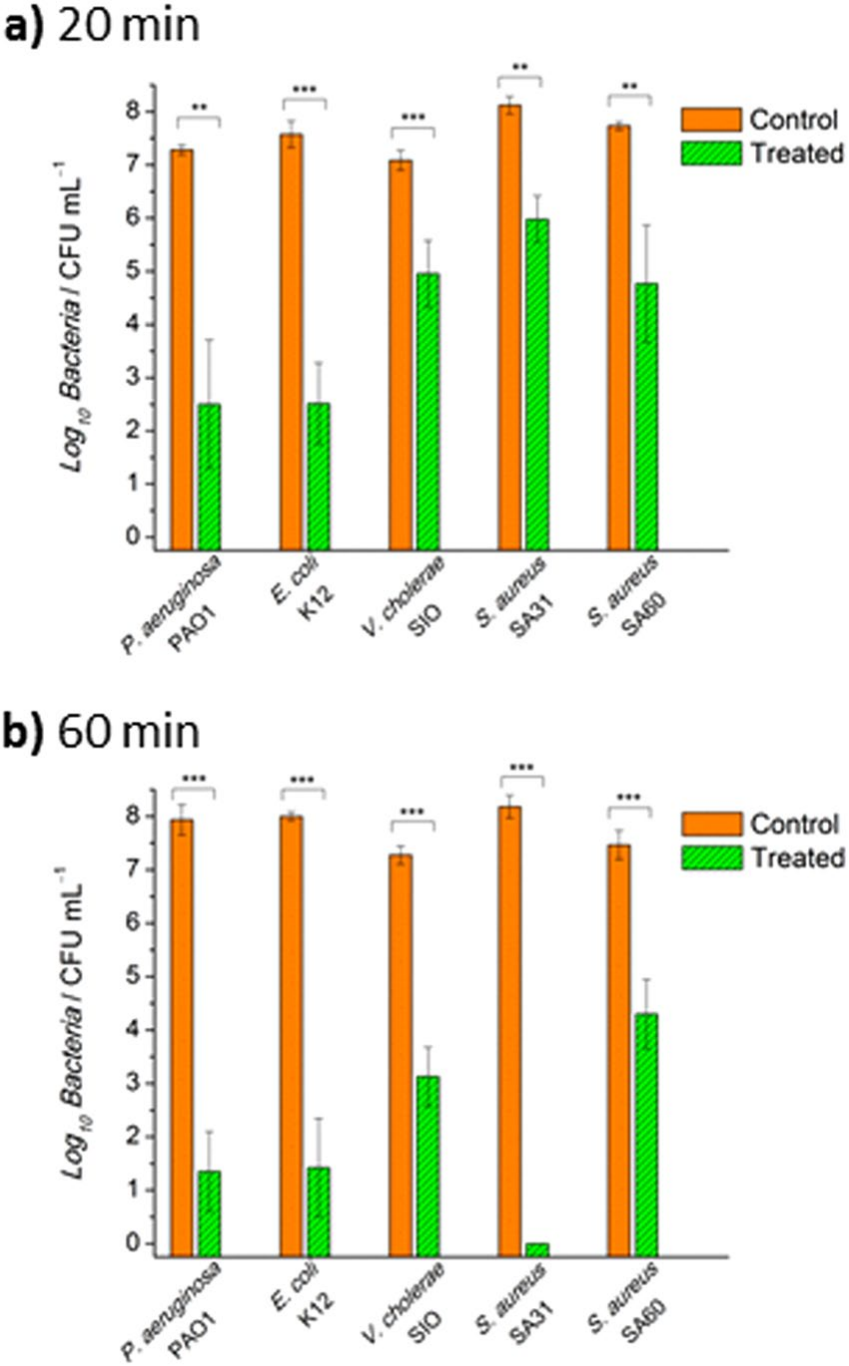
Broad spectrum antimicrobial activity. Colony forming unit (CFU) analysis of various bacteria-contaminated bulk water following 20 min (**a**) and 60 min (**b**) of treatment with macroporous antimicrobial polymeric gel (MAPG). All data are expressed as mean ± s.d. (as indicated by error bars) based on values obtained from at least 3 biological replicates (n ≥ 3). Student’s *t*-test, **p < 0.01, ***p < 0.001 showing significant difference between the control (untreated) and treated data.

The number of viable bacteria reduced in the bulk water after treatment with MAPG for 20 and 60 min is summarized in Fig. 2 and Table 1. After 20 min of treatment, >2.0 log_10_ reduction in CFU was observed for all the bacteria tested, which translates to >99% of viable bacteria removed from the bulk water. Longer incubation time of 60 min resulted in greater reduction in CFU. For *S. aureus*, no viable bacteria were detected in the bulk water (i.e., >8.2 log_10_ reduction in CFU) whereas for Gram-negative bacteria, >4.0 log_10_ reduction in CFU was achieved after 60 min of treatment. MAPG was also effective against clinical MRSA strain, removing >99.9% of the live pathogen from the bulk water after 60 min of treatment. The antimicrobial performance of MAPG is extremely efficient and impressive in terms of the amount of bulk water it can disinfect, the broad-spectrum activity, the log_10_ reduction in CFU and the treatment time, as compared to other antimicrobial polymer gels in literature^16,17,19^. In fact, the reported antimicrobial polymer gels are only effective in disinfecting small volumes of water absorbed by the gel, not the bulk water, which limits their potential applicability to biomedical devices.

The antimicrobial performance of MAPG was also compared to HG where slices of HG were subjected to the same antimicrobial test in a 24-well plate system using *P. aeruginosa* and *S. aureus* as model bacteria. CFU analysis of the bulk water after treatment with HG for 60 min showed only 37.5% and 96.8% reduction of live *P. aeruginosa* and *S. aureus*, respectively (Fig. S3, SI and Table 1). The activity of HG is significantly poorer than MAPG. This is not surprising as the porous structure of MAPG provides a high surface area for efficient bacteria adsorption which is not available in HG. This clearly demonstrated the advantage of macroporous gels over traditional hydrogels for antimicrobial applications.

An additional experiment was also performed to determine if MAPG is still feasible in a larger scale operation. For this, a 6-well plate system was used where the size of each well is larger than the 24-well plate. The weight of MAPG and the volume of bacteria-contaminated water used for this experiment were scaled up accordingly. The experiment was performed against *E. coli* (ca. 10^8^ CFU mL^−1^). CFU analysis revealed that MAPG is still very effective when scaled up and resulted in ca. 3.0 log_10_ and 4.0 log_10_ reductions after 20 and 60 min of treatment, respectively, even though the effectiveness of the 6-well plate system is lower than the 24-well plate system by ca. 2.0 log_10_ reduction in CFU (Fig. S4, SI).

### Recyclable Antibacterial Syringes

**Figure 3:**
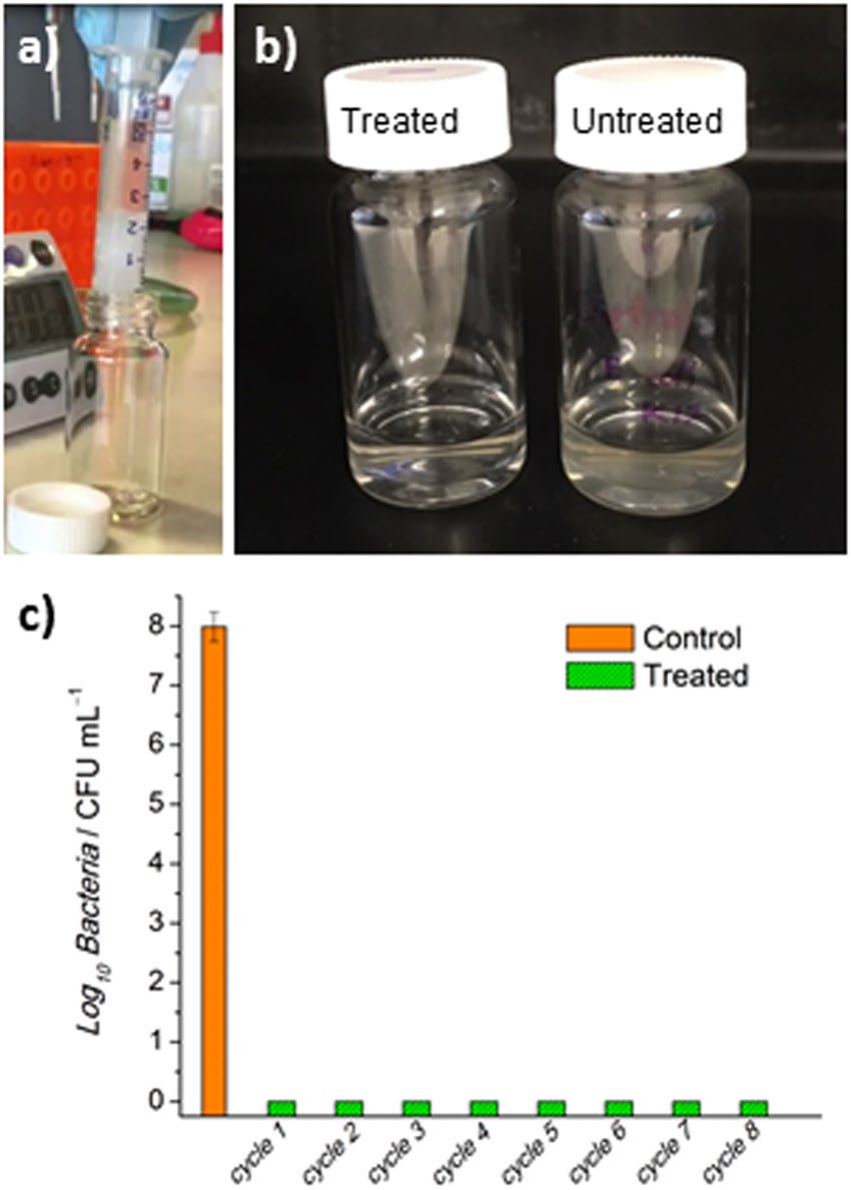
Recyclable bacteria disinfecting syringes. (**a**) Image of macroporous antimicrobial polymeric gel (MAPG) in a syringe and *E. coli*-contaminated water passing through it. (**b**) Image of treated water after passing through the syringe. Compared to the untreated water which is cloudy because of the presence of bacteria, the treated water is clear. (**c**) The syringe was subjected to 8 continuous cycles of percolation with *E. coli*-contaminated water where the recovered water were analysed via colony forming unit (CFU) analysis. No viable bacteria were detected in all the water that passed through the syringe.

Given the excellent antibacterial properties of free-standing MAPG in a well-plate system, we next tested the efficacy of MAPG in a syringe format similar to a column chromatography setup (Fig. 3a). The synthesis of MAPG in the syringe proceeded in the same fashion as before. For testing the antimicrobial performance of MAPG syringes, *E. coli* was used as the model bacteria. Like in the earlier experiments, the bacteria concentration was kept at ca. 10^8^ CFU mL^−1^. The bacteria solution was percolated over the syringe at a flowrate of ca. 1 mL min^−1^, and the treated water that exited the syringe was subjected to CFU analysis. Visual inspection clearly showed that the treated water is clear (Fig. 3b). In fact, no viable bacteria were detected in the treated water by CFU analysis, which indicated >8.0 log_10_ reduction in bacteria counts compared to the untreated controls (Fig. 3c). The recyclability of the syringe was investigated by subjecting it to multiple passes of *E. coli*-contaminated water for a total of 8 cycles. Remarkably, no viable bacteria were detected in all percolated, treated water samples while visual inspection of the samples showed the waters were clear (Fig. 3c and Fig. S5, SI). In total, the MAPG syringe disinfected 24 mL of bacteria-contaminated water, which is >120 times the mass of the organic material used, with no detectable viable bacteria in all collected fractions. The syringe system outperformed the well-plate system. This is most likely because the contaminated water is forced to fully interact with the gel via a unidirectional movement in the syringe system, as opposed to the well-plate system where contact between the bacteria and the gel is purely based on random Brownian motion. In essence, the contact between bacteria and gel is not as ordered and efficient in the well-plate system than in the syringe system. Nonetheless, it is worth emphasizing that the antimicrobial activity of the well-plate system is still excellent. The results from both the well-plate and syringe systems thus strongly indicate that good contact between bacteria and gel is vital for high antimicrobial performance.

### Antimicrobial Mechanism Investigation

**Figure 4:**
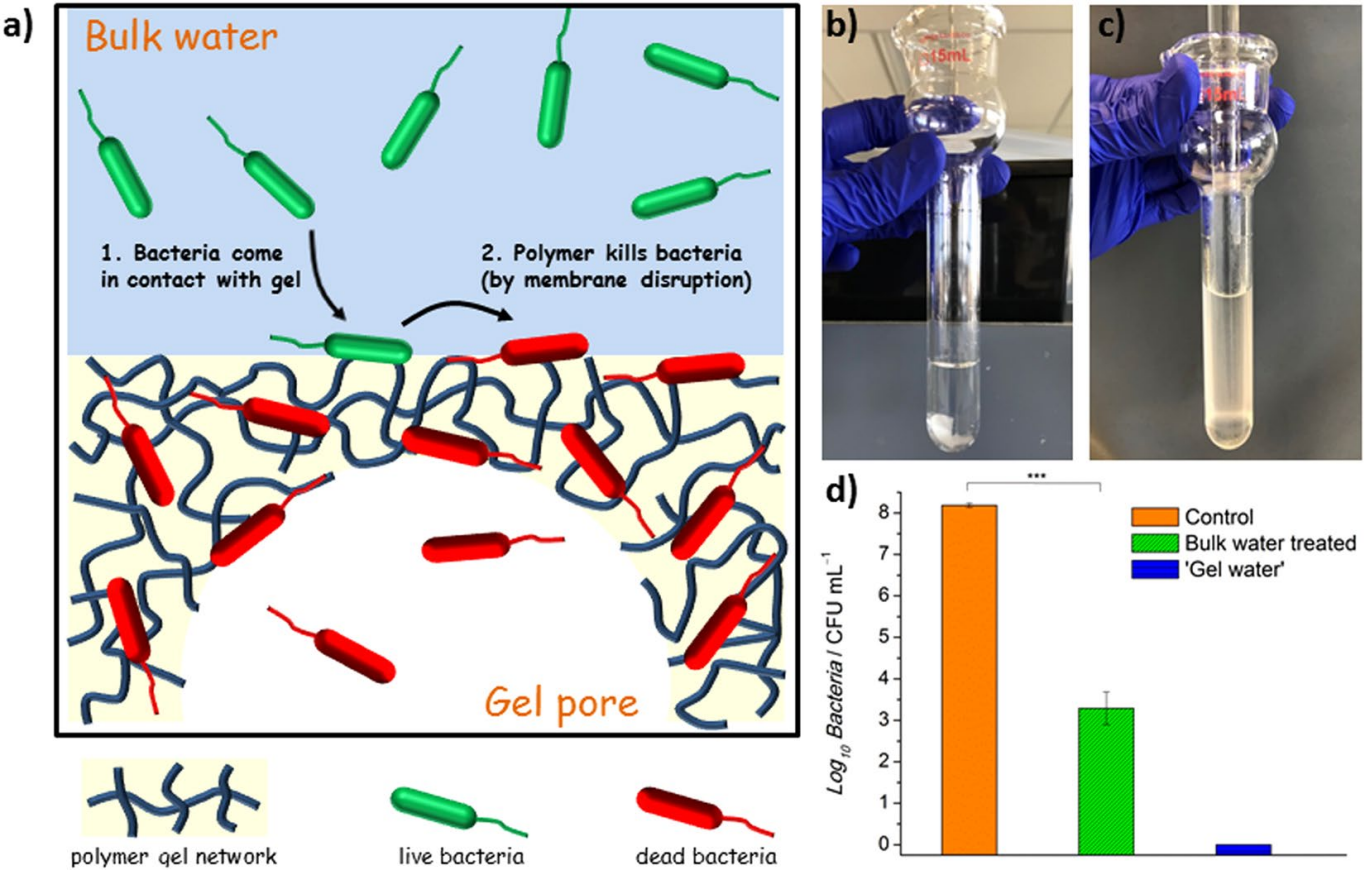
Antibacterial mechanism of macroporous antimicrobial polymeric gel (MAPG). (**a**) Illustration depicting the bacterial disinfection mechanism of MAPG in bacteria-contaminated water. (**b**) Image of MAPG after it was used in the disinfection of *E. coli*-contaminated water. The gel was resuspended in fresh phosphate buffered saline (PBS). (**c**) Image of crushed MAPG slurry in PBS after the gel was thoroughly grinded with a pestel. (**d**) Colony forming unit (CFU) analysis of the crushed gel slurry compared to the gel-treated bulk water. All data are expressed as mean ± s.d. (as indicated by error bars) based on values obtained from at least 3 biological replicates (n ≥ 3). Student’s *t*-test, **p < 0.01, ***p < 0.001 showing significant difference between the control (untreated) and treated data.

Our initial hypothesis on the antimicrobial mechanism of MAPG is that the bacteria are first adsorbed onto the material surface, followed by subsequent inactivation by the polymers (Fig. 4a). The overall mechanism therefore procedes via contact active mode. In the abovementioned results, the CFU analysis data of the bulk water that has been treated in the well-plate and syringe systems can only confirm the amount of viable bacteria that were still present in the treated water samples, but do not reveal the fate of those that have been adsorbed by the gel. If the proposed mechanism is correct, the majority of the bacteria adsorbed by the gel should be dead. To prove this, a sample of MAPG that has been used in the disinfection of *E. coli*-contaminated water in a well-plate system was crushed with a tissue-grinder apparatus (Fig. 4b,c), and the contents were analyzed by CFU analysis to determine the viability of the bacteria that were adsorbed by the gel. This method of determining the bacteria cell viability is comparable to standard procedures of determining the bacteria content in tissue samples for *in vivo* experiments. It is noteworthy that the bulk water was removed prior to the crushing of MAPG. No viable bacteria were detected in the crushed gel aqueous sample, which indicated that all the bacteria that were adsorbed by the gel were dead. This verifies the proposed mode of action of MAPG (Fig. 4d). An additional experiment was also performed to demonstrate that the antimicrobial activity of MAPG was not due to the leaching of polymers into solution which then inactivate the bacteria. Using a well-plate system, MAPG was incubated in fresh phosphate buffered saline (PBS) solution under identical conditions to an antimicrobial susceptibility assay, and the solution was then mixed with *E. coli*-contaminated water to determine if the solution contained any leached polymers which may be responsible for the perceived antimicrobial properties. CFU analysis revealed there was no significant difference between the untreated *E. coli*-contaminated water with the ones that have been mixed with MAPG-incubated solutions. This confirmed that the antimicrobial activity of the gel is indeed exerted by the bulk material itself.

## Discussion

The development of polymer gels and scaffolds with inherent antimicrobial properties is an emerging field as these materials offer new avenues in combating (multidrug-resistant) bacteria. In this study, we demonstrated the fabrication of a novel material termed macroporous antimicrobial polymeric gel (MAPG) that exhibits excellent inherent antimicrobial activity against a range of Gram-negative and Gram-positive bacteria as a proof-of-concept for point-of-use water disinfection application. The ability to disinfect a wide range of bacteria family is an important criteria for water disinfection technology as water systems contain mixture of microbes. From a synthetic point of view, MAPG is completely organic and is easily produced at subzero temperature via free radical polymerization of monomers and initiators which can be bought commercially or made in a facile manner at multigram scale. This suggests that the material production process could be implemented by industry.

We also demonstrated that MAPG can disinfect bacteria-contaminated water (>50 times the mass of the organic material used) and effectively inactivates >99% of bacteria from the bulk water within 20 min of treatment. Furthermore, we showed that MAPG can also be formulated into a syringe which can be reused multiple times without losing potency. The MAPG syringe can disinfect bacteria-contaminated water that is >120 times the mass of polymer used with an inactivation efficiency of >8.0 log_10_ reduction in CFU compared to the untreated controls. The overall antimicrobial performance of MAPG is truly impressive compared to literature reported materials (especially bearing in mind that a high bacteria concentration was applied, ca. 10^8^ CFU mL^−1^), and we showed that this is due to the combination of specific amphiphilic cationic polymers used and the macroporous structure of the gel. The capabilities to disinfect water with high bacteria loading and to reuse multiple times are essential for a device to be applied in a practical sense. Although the antimicrobial performance of MAPG is truly remarkable, additional studies are needed in the future to further ascertain the limit of (next-generation) MAPG. In addition, the organic matter content in bacteria-contaminated water needs to be considered for potential real world applications given that MAPG contains hydrophobic groups which may bind with these organic compounds. Another interesting aspect of MAPG is that this technology could potentially be extended to other fields, for instance, as wound healing materials. The macroporous structure of MAPG can potentially be used to load specific biomolecules such as growth factors to be delivered to wound sites whilst the MAPG scaffold itself eliminates any bacteria infection – all in all accelerating the healing process. We envisage that the platform technology of MAPG will find real world applications in point-of-use water disinfection as well as in other related bacteria inactivation applications.

## Methods

### Materials

1-Bromohexane (Aldrich, 98%), 2-(dimethylamino)ethyl methacrylate (DMAEMA) (Aldrich, 98%), oligo (ethylene glycol) dimethacrylate (OEG-DMA) *M*_n_ 550 g mol^−1^ (Aldrich, 98%), ammonium persulfate (APS) (Sigma-Aldrich, 98%), *N,N,N′,N″,N″-*pentamethyldiethylenetriamine (PMDETA) (Aldrich, 99%), chloroform (CHCl_3_) (VWR Chemicals), acetonitrile (ACN) (VWR Chemicals) and diethyl ether (DEE) (Merck) were used as received. Milli-Q water with a resistivity of >18 MΩ⋅cm was obtained from an in-line Millipore RiOs/Origin water purification system.

### Instrumentation

^1^H Nuclear magnetic resonance (NMR) spectra were attained using a Bruker AC300F spectrometer. Deuterated solvent D_2_O (obtained from Cambridge Isotope Laboratories) was used as reference solvent. The sample concentration was ca. 20 mg mL^−1^. The surface morphology of swollen gels was investigated using FEI Quanta 200 F SEM at an accelerating voltage of 10 keV. Samples were directly mounted on carbon tape and were coated with a thin layer of gold.

### Synthesis of Quarternary Ammonium (QA) Monomer

To a round-bottom flask that contained 1-bromohexane (10.7 mL, 0.153 mol) in 75 mL of ACN:CHCl_3_ (2:1 volume ratio) solvent mixture, was added DMAEMA (10.7 mL, 0.127 mol). The reaction mixture was stirred at 40 °C for 16 h. The contents were poured slowly into a beaker of cold DEE (2 L) to precipitate the product. The filtrate was collected, washed with DEE (ca. 1 L), and dried in vacuo for 24 h to yield the pure QA monomer as a white solid. The yield was 18 g (0.056 mol). The purity of the monomer was confirmed by NMR spectroscopy (Fig. S1, SI).

### Gel Fabrication

QA monomer (1.7 mmol, 550 mg) and OEG-DMA (0.17 mmol, 93.3 mg) were prepared in a 7 mL glass vial followed by the addition of Milli-Q water (1.5 g) for dissolution of the contents. Then, PMDETA (68 μmol, 14.1 μL) was added to the solution. The redox initiator APS (34 μmol, 7.7 mg) was added last and the solution was mixed thoroughly. The glass vial was subsequently sealed. The freshly prepared polymerizable media was immediately frozen in liquid N_2_ and kept in a freezer at −20 °C for 3 days to obtain MAPG. To obtain HG, the solution was left to stand at room temperature for 3 h. The glass vial that housed the gel was gently broken to recover the gel. The gels were purified by immersing in Milli-Q water (100 mL) for 10 h which included periodic changing of the solvent every 2 h. To obtain MAPG in a syringe, the polymerizable media (ca. 2 g) was prepared in a 6 mL plastic luer-lock sterile syringe and immediately frozen in liquid N_2_ and kept in a freezer at −20 °C for 3 days. The gel in the syringe was purified in the same manner as above.

### Mechanical Testing

The mechanical properties of the swollen gel samples (10 mm thickness, and diameter ~9 mm) were characterized using Zwick Roell Z-10-UTM at 25 °C. The load was adjusted to 5 kN and a strain rate of 5 mm min^−1^ was used, and the sample was compressed up to 40% strain of its initial length. Experiments were conducted in triplicate. The experimental parameters are listed in Table S1, SI.

### Swelling Degree Studies

The swelling properties were determined by gravimetric analysis. For the determination of the swelling profile, the gel samples were lyophilized until a constant weight. Dried gels were allowed to swell in excess of water and the water uptake by the gels was determined by measuring the cumulative mass increase at a predetermined time interval. It is worthwhile noting that excess surface water was gently wiped off with tissue paper before measuring the mass of the swollen gel. The degree of swelling at time *t* was calculated as a ratio of *M*_*t*_/*M*_0_ where *M*_*t*_ and *M*_0_ are the masses of the swollen gel at time *t* and dried gel, respectively. The experiment was done in triplicate.

### Bacteria Cell Culture

In all assays, glycerol stock of *Pseudomonas aeruginosa* PAO1, *Escherichia coli* K12, *Vibrio cholerae* SIO, *Staphylococcus aureus* SA31, or methicillin-resistant *S. aureus* SA60 was streaked onto Luria-Bertani (LB) medium agar plate and cultured overnight at 37 °C. Afterwards, a single colony of bacteria was picked and inoculated in 10 mL of LB medium at 37 °C with shaking at 180 rpm overnight. The overnight culture was diluted 1:100 in 10 mL of LB and allowed to grow to mid log phase at 37 °C with shaking at 180 rpm for ca. 2.5–3 h, then further diluted 1:5 in phosphate buffered saline (PBS) media (pH 7.4) for subsequent antimicrobial assays. The bacteria concentration in the PBS solution was ca. 10^8^ CFU mL^−1^.

### Antimicrobial Assay for Well-Plate System

Fully swollen gels were cut into thin slices of ca. 15 × 2 mm (ca. 200 mg) and each placed into a well of a 24-well plate. Noteworthy, the mass of the organic content per slice (excluding the absorbed water) was ca. 20 mg for MAPG. To each well was added 1 mL of the prepared bacteria solution in PBS, and the plate was incubated at 37 °C with shaking at 180 rpm for 20 or 60 min. After treatment, bacteria cell viability analysis of the treated bulk water was determined by a drop plate method. The bulk water was serially diluted (10-fold) in sterile PBS and plated onto LB agar plate. Colonies were counted and CFU analysis was determined after the plate was incubated for 24 h at 37 °C. All assays included two replicates and were repeated in at least three independent experiments.

### Antimicrobial Assay for Syringe System

With the syringe system, 3 mL of the prepared bacteria solution in PBS was added onto the top of the MAPG syringe. The liquid was plunged through the syringe manually at a flowrate of ca. 1 mL min^−1^, and the water that exited the syringe was analyzed by CFU analysis as abovementioned. The MAPG syringe was recycled consecutively for a total of 8 times using fresh bacteria solution. The breakthrough volume is ca. 240 mL g^−1^ of polymer material. This assay included two replicates and was repeated in three independent experiments.

### Antimicrobial Mechanism Investigation

A slice of the MAPG gel, which was used in the 60 min treatment of *E. coli*-contaminated water in a well plate system, was washed twice with PBS and subsequently placed in a 15 mL Dounce tissue grinder (Sigma-Aldrich), followed by the addition of PBS (5 mL). The gel was thoroughly crushed with a pestel until a slurry is formed. The mixture was homogenized in PBS by incubating in an ultrasonication bath (150 W, 40 Hz) at room temperature for 2 min. The mixture was then subjected to the same CFU analysis procedure. This assay was repeated in three independent experiments.

### Statistical Analysis

One-way classification of ANOVA and student’s t-test (two-tailed) were performed where differences between data were regarded as statistically significant with *p*-values < 0.05.

## Electronic supplementary material

Supplementary Information
