# Supplementary material for: Highly Bactericidal Macroporous Antimicrobial Polymeric Gel for Point-of-Use Water Disinfection
Source: Sci Rep. 2018 May 21;8:7965. doi: 10.1038/s41598-018-26202-0 (PMC5962547; doi:10.1038/s41598-018-26202-0)
Supplement: Supplementary file 1 — Supplementary Information [file 41598_2018_26202_MOESM1_ESM.pdf]

Supplementary Information

Highly Bactericidal Macroporous Antimicrobial Polymeric Gel for Point-of-Use Water Disinfection

Amit Kumar[1], Cyrille Boyer[2], Leena Nebhani[1,*] & Edgar H. H. Wong[2*]

[1]Department of Materials Science and Engineering, Indian Institute of Technology Delhi, Hauz Khas, New Delhi 110016, India
[2]Centre for Advanced Macromolecular Design (CAMD) and Australian Centre for NanoMedicine (ACN), School of Chemical Engineering, UNSW Australia, Sydney, NSW 2052, Australia

Correspondence and requests for materials should be addressed to E.H.H.W. and L.N. (email: edgar.wong@unsw.edu.au, leena.nebhani@polymers.iitd.ac.in)

Keywords: water treatment, bacteria, functional polymer, polymerization, advanced materials

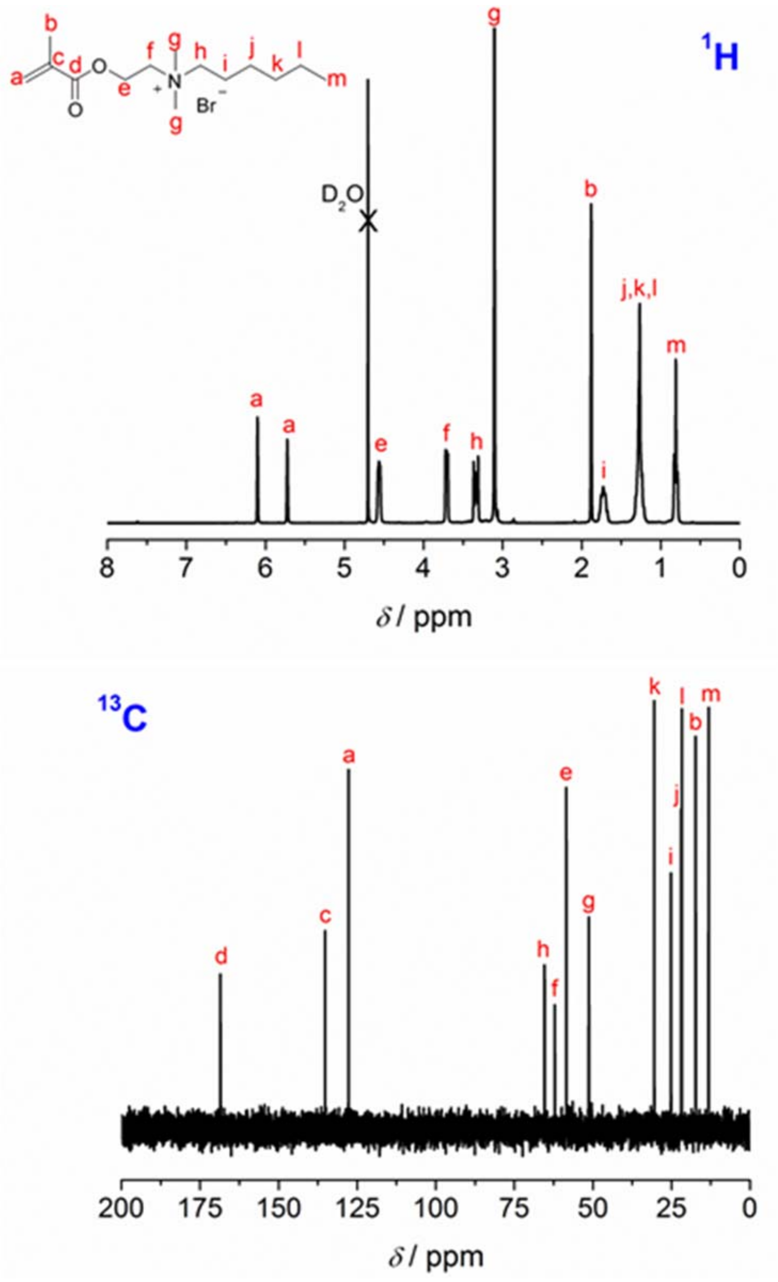

Figure S1. NMR analysis of QA monomer. ^{1}H NMR (top) and ^{13}C NMR (bottom) spectra of synthesized QA monomer in D_2O.

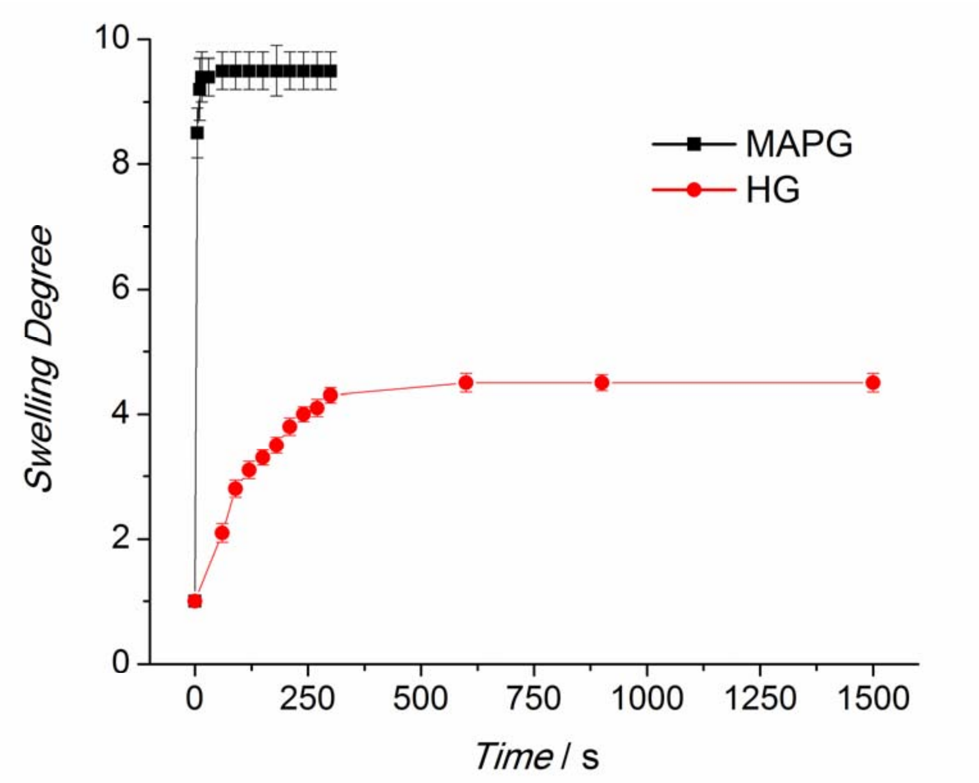

Figure S2. Swelling degree of gels. Equilibrium is reached after a certain time period and is different for both gel types.

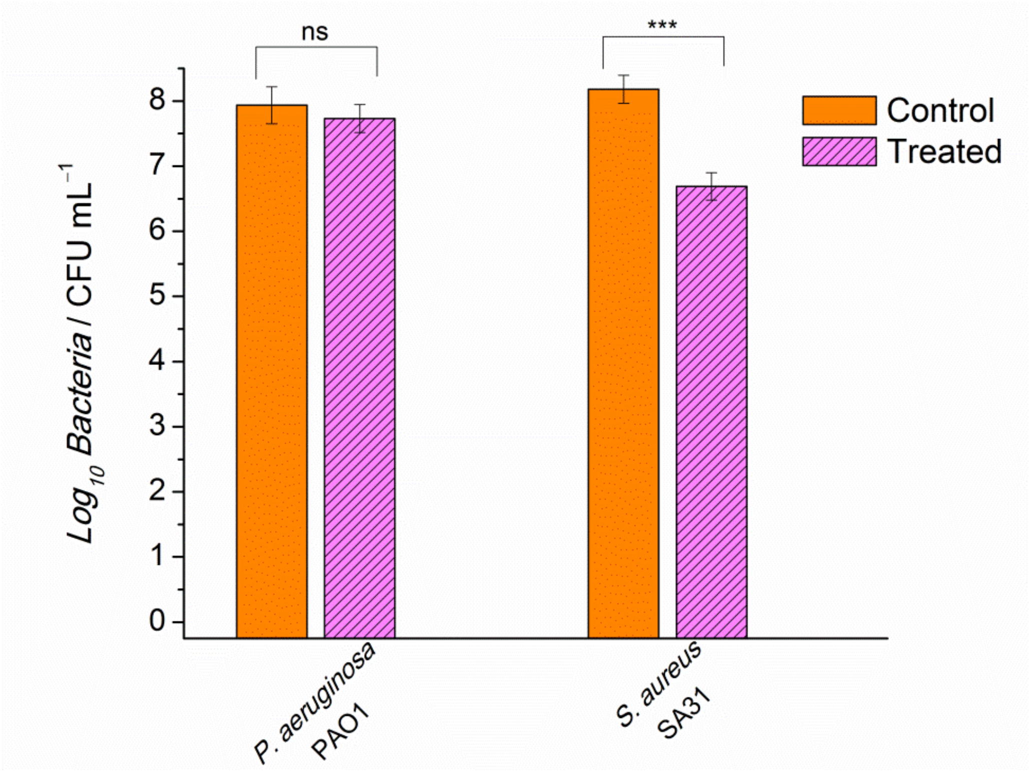

Figure S3. Antimicrobial activity of HG. CFU analysis of bacteria-contaminated water following 60 min of treatment with hydrogels. All data are expressed as mean ± s.d. (as indicated by error bars) based on values obtained from at least 3 biological replicates ($n \geq 3$). Student's *t*-test, ns>0.05, ****p*<0.001 showing (in)significant difference between the data.

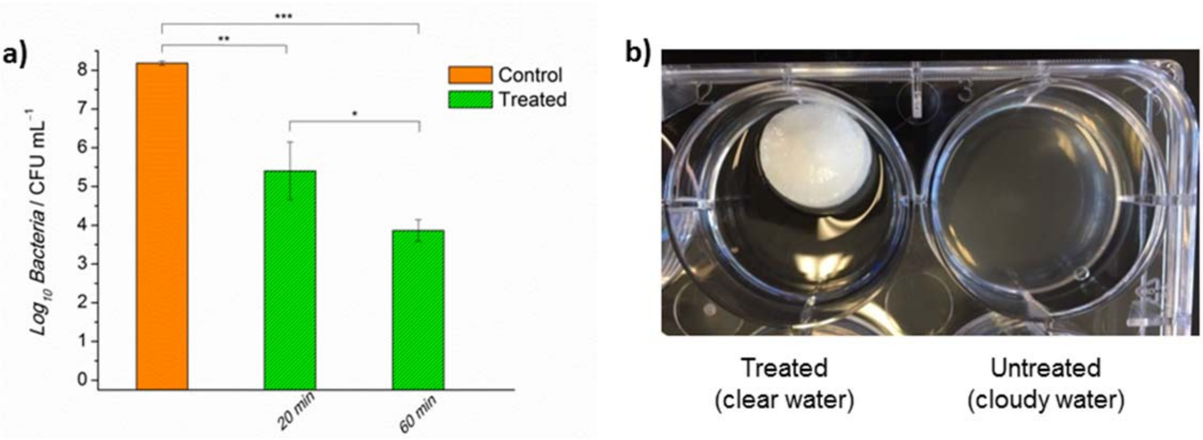

Figure S4. Antimicrobial performance of MAPG in a larger scale operation. (**a**) CFU analysis of *E. coli*-contaminated water following 20 min and 60 min of treatment with MAPG in a 6-well plate system. All data are expressed as mean ± s.d. (as indicated by error bars) based on values obtained from at least 3 biological replicates (n ≥ 3). Student's *t*-test, *$p<0.05$, **$p<0.01$, ***$p<0.001$ showing significant difference between the data. (**b**) Image of the well plate taken after 60 min of incubation. The water treated with MAPG is clear whereas the untreated water is cloudy due to the presence of bacteria (ca. 10^8 CFU mL^{-1}).

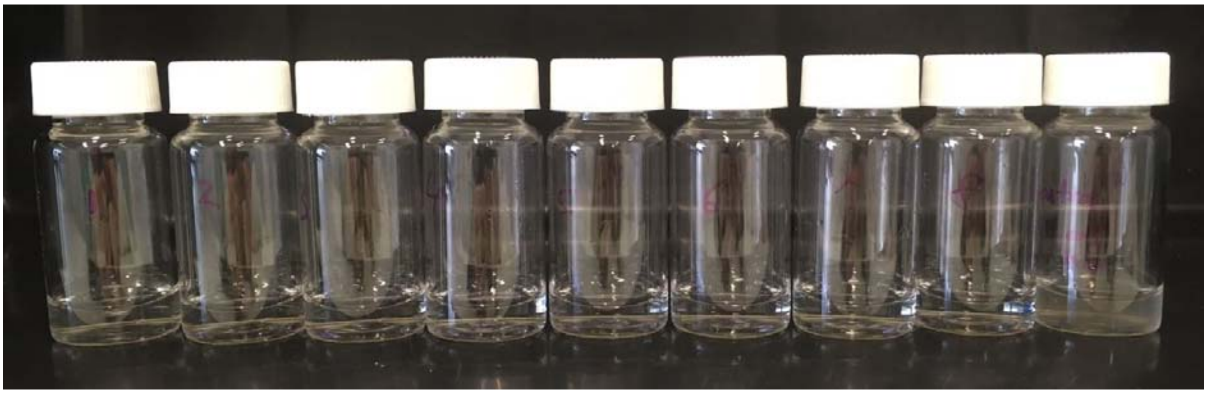

Figure S5. Water treated by MAPG syringe. Images of obtained clean and clear water after *E. coli*-contaminated water was passed through the same MAPG syringe multiple times. The fractions in ascending order (1 to 8) are from left to right while the untreated water, which is cloudy, is on the far most right.

Table S1. Experimental parameters for mechanical compression testing of gels.

Gel	Maximum Force / kPa	Strain at maximum force / %	*E* / kPa
MAPG	280 ± 90	24.5 ± 2.1	567 ±74
HG	160 ± 80	19.5 ± 1.3	683 ± 42
